# Psychometric properties of the patient assessment of chronic illness care measure: acceptability, reliability and validity in United Kingdom patients with long-term conditions

**DOI:** 10.1186/1472-6963-12-293

**Published:** 2012-08-31

**Authors:** Jo Rick, Kelly Rowe, Mark Hann, Bonnie Sibbald, David Reeves, Martin Roland, Peter Bower

**Affiliations:** 1 Centre for Primary Care Research, Institute of Population Health, Manchester Academic Health Science Centre (MAHSC), University of Manchester, Manchester, UK; 2 Directorate for the Student Experience, University of Manchester, Manchester, UK; 3 Cambridge Centre for Health Services Research, University of Cambridge, Cambridge, UK

**Keywords:** Long term conditions, Chronic disease, Patient assessments, Primary care, Quality improvement

## Abstract

**Background:**

The Patient Assessment of Chronic Illness Care (PACIC) is a US measure of chronic illness quality of care, based on the influential Chronic Care Model (CCM). It measures a number of aspects of care, including patient activation; delivery system design and decision support; goal setting and tailoring; problem-solving and contextual counselling; follow-up and coordination. Although there is developing evidence of the utility of the scale, there is little evidence about its performance in the United Kingdom (UK). We present preliminary data on the psychometric performance of the PACIC in a large sample of UK patients with long-term conditions.

**Method:**

We collected PACIC, demographic, clinical and quality of care data from patients with long-term conditions across 38 general practices, as part of a wider longitudinal study. We assess rates of missing data, present descriptive and distributional data, assess internal consistency, and test validity through confirmatory factor analysis, and through associations between PACIC scores, patient characteristics and related measures.

**Results:**

There was evidence that rates of missing data were high on PACIC (9.6% - 15.9%), and higher than on other scales used in the same survey. Most PACIC sub-scales showed reasonable levels of internal consistency (alpha = 0.68 – 0.94), responses did not demonstrate high skewness levels, and floor effects were more frequent (up to 30.4% on the follow up and co-ordination subscale) than ceiling effects (generally <5%). PACIC demonstrated preliminary evidence of validity in terms of measures of long-term condition care. Confirmatory factor analysis suggested that the five factor PACIC structure proposed by the scale developers did not fit the data: reporting separate factor scores may not always be appropriate.

**Conclusion:**

The importance of improving care for long-term conditions means that the development and validation of measures is a priority. The PACIC scale has demonstrated potential utility in this regard, but further assessment is required to assess low levels of completion of the scale, and to explore the performance of the scale in predicting outcomes and assessing the effects of interventions.

## Background

Improving the quality of care for long-term conditions is an international priority [1,2], which has led to significant focus on the delivery and evaluation of quality improvement activities such as provider and patient education [1], service redesign [3], use of technology [4], and financial incentives [5]. However, assessing the effects of these initiatives depends on acceptable, reliable and valid measures of quality. Although quality can be assessed from a number of perspectives, there is increasing agreement concerning the importance of the views of the patient [6].

Assessing the patient perspective generally requires self-report measures. To ensure their utility, measures must be subject to a significant programme of research to assess their acceptability to patients and their formal psychometric properties, including their use in contexts and populations different to those in which they were developed. In particular, where innovations in the management of long-term conditions cross national boundaries, measures of quality are needed that perform consistently in different health care settings to both support effective local policy implementation and to allow interpretable comparison of the performance of different health care systems worldwide.

The Patient Assessment of Chronic Illness Care (PACIC) is a United States (US) measure of quality of care for patients with a chronic illness [7]. The original PACIC includes 20 items and measures specific actions or qualities of care, based on the influential Chronic Care Model (CCM). The PACIC is designed around five subscales: (a) patient activation (b) delivery system design and decision support (c) goal setting and tailoring (d) problem-solving and contextual counselling (e) follow-up and coordination.

Although the scale was only published in 2005, the influence of the Chronic Care Model means that there is already a reasonable evidence base on the performance of the scale (see Table 1). The scale seems to be largely acceptable to patients with long-term conditions, with low levels of missing data [7-10], although some studies demonstrate skew and related floor and ceiling effects [11,12]. Most assessments suggest acceptable levels of internal consistency [7,9,11,13-15] and test-retest reliability [7,13,15]. Although the scale is based on a five factor conceptual model, there is less consensus over the degree to which responses reflect this structure [9,11], with studies suggesting two factor or unidimensional structures may be a better fit to the data [8,9,14].

**Table 1:** Summary of published validity data on the PACIC

**Author**^**1**^	**Country**	**Mean scores (&**		**Acceptability**	**Structure**	**Associations with:**	**Associations with:**
**PACIC version**	**N (Response rate)**	**Cronbach’s alpha)**				·**health conditions**	·**other measures**
	**Context**	**a. PACIC; b. PA;**				·**other measures of**	·**patient characteristics**
		**c. DS/PD; d. GS/T; e. PS/C; f. F/C (other reliability data)**				**chronic care**	·**interventions**
Aragones [15]	USA	a.3.17 (0.87)		Reports no ceiling or floor effects	Type of analysis not clear	No significant association with number of chronic conditions	No significant association with age, sex, education, insurance, years in the US
Spanish language version	Sample 1: 100/120 (83%)	b. – f. 2.50 – 3.95 (all >0.6)			Factor loading analysis – most items correlated highly on proposed scales		
	Sample 2: 20 telephone interview follow ups						
	Spanish speaking Hispanics with diabetes in hospital ambulatory settings	(Test Re-test 0.77)					
Carryer [16]	New Zealand	GP care/Nurse care					Professional self ratings much higher (on modified version of PACIC):
Modified PACIC for professionals	Sample 1: 341 (85.3% - of those who expressed an interest in participating)	a.2.7/3.3					a.4.0
		b.2.9/3.5					b.4.3
		c.3.1/3.7					c.3.8
		d.2.3/3.2					d.3.8
	Sample 2: 89 GPs & nurses	e.2.8/3.5					e.4.1
	Primary care patients and practitioners	f.2.6/2.9					f.3.8
Gensichen [8]	Germany	a.3.25 (0.91)		Ceiling effects: PA (12.9%) and PS/C (8.9%)	EFA two factors (‘Patient activation	Overall PACIC with number of conditions and PHQ9 both NS	No significant associations with age, sex, education
German language version	442/485 (91.1%)	b.3.65 (0.80)		Floor effects:	and problem solving’ and ‘ Goal setting and co-ordination’) 46.5%	High correlations with all EUROPEP scales	
	Patients with major depression in primary care	c.3.47 (0.45)		GS/T (4.6%)			
		d.2.97 (0.74)		Missing data from 0.7% - 5.4%	some items did not load as expected		
		e.3.69 (0.77)					
		f.2.83 (0.76)					
Glasgow [7]	USA	a.2.60 (0.93)		No items had ceiling effects	CFA – moderate fit	No variation in response across 6 most common long- term conditions (excluding diabetes patients who report better follow up); Higher PACIC scores associated with more conditions (r = 0.13, p<0.05)	Correlations (PACIC and subscales) with patient characteristics all <=0.25; Higher overall PACIC related to age (higher) and gender (female); Gender significantly related to all subscales (0.14 to 0.25; P<0.05)
	Sample 1: 379/500 (76%) of which 283 had chronic condition (57%)	b.2.99 (0.82)		Floor effects identified, but not reported in detail			
		c.3.13 (0.77)		96% had no missing data			
		d.2.43 (0.84)					
		e.2.87 (0.90)					
	Sample 2: 82/100 sent follow up at +12 weeks (82%) of which 63 had chronic condition (63%)	f.1.07 (0.86)					
	Primary care	(3 month re-test 0.58)				Overall PACIC and all subscales correlate significantly with Hibbard Activation and Safran Assessment of primary Care sub scales (with exception of PACIC F/C and Safran Integration sub scale)	
Glasgow [12]	USA	a.3.2 (0.96)		Adequate variability		No significant relationship to number of conditions	Correlated with physical activity (r=0.17) but not fat consumption
Includes PACIC 5As	363 (63%)	b.3.6		3-9% sub scale scores <1.5, (4% on summary scale)		Related to quality of care (composite lab assessment r=0.23) and composite self management support (r=0.25)	No significant differences with sex, ethnicity or income
	Type 2 diabetes patients in primary care	c.3.5		7-22% sub scale scores >4.5 (9% on summary scale)			
		d.3.0					
		e.2.9					
		f.3.4					
		5As mean = 3.2					
Goetz [17]	Germany			Patients tended to gravitate to both end points (0% and 100%)	FA indicated a 1 factor solution for the PACIC short form	There was no correlation between the mean overall score of the PACIC short form and number of chronic conditions	
PACIC short form & revised scoring	264 (49%)			Non-response rates ranged from 4.2% - 12.5%			
	Over 18 with at least one chronic condition in primary care						
Gugiu [13]	US	(Short form PACIC – 11 items – Ordinal alpha = 0.955 (sample 1) and 0.963 (sample 2); Ordinal omega 0.956 (S1) & 0.963 (S2); Eight month Test re-test reliability (n=250) = 0.638)			EFA within a CFA	No associations with HBa1c, LDL, microalbumin, BP	
Modified PACIC percentage scale	Sample 1: 529/943 (55%)				Unidimensional, 11 item variant		
	Sample 2: 361/943 (38%) (111 not in first sample)						
	Type 2 diabetics, large physician networks						
Gugiu [9]	USA	(Short form PACIC – 11 items, Alpha 0.945, ordinal alpha 0.972, ordinal omega 0.973)		Missing data 0.2%	CFA Poor fit to 5 factors	No associations with clinical indicators	
Modified PACIC percentage scale (linked to above)	539/943 (57%)			to 2.8%, 8.9% failed to respond to at least 1	EFA 1–3 factors, 1 factor preferred		
	Type 2 diabetics, large physician networks			Kurtosis (trimodal, 43% 90-100%, 24% 0-10%)			
Jackson [18]	USA	a.3.1					Non white patients more likely to report experience consistent with the CCM (OR 2.3) (PS and FU significant among subscales); Patients not completing high school more likely to report experience consistent with the CCM (OR 3.0) and subscales
	204 (69%), but 189 (64%) complete information	b.3.3					
	Patients with diabetes receiving VA primary care services	c.3.6					
		d.3.1					
		e.3.4					
		f.2.6					
Maindal [11]	Denmark	a.(0.94)		Missing 0.5 – 2.9%	CFA good fit for 2 indices, poor for 4	Patients with self-rated good health reported higher scores on ‘Patient Activation’, ‘Decision Support’ and ‘Goal Setting’; Patients with more than one additional disease rated lower on PA and DS	No significant associations with sex, age
Danish Language version	1265/2476 but only 560 met criteria of diabetes > 2 years + medical treatment (22.6%)	b. – f. (0.71 – 0.86)		Floor effects: 2.7% - 69.2%, >15% for 17 items			
	Patients on national diabetes register			Ceiling effects: 4.0% – 4.04%, >15% for 12 items			
Rosemann [19]	Germany	Male/Female		Adequate variability	Education and age predicted overall PACIC score in regression	Significant relationships with disease duration, BMI, co-morbidities, PHQ sum, AIMS2F,	Significant differences by gender and educational level (p<0.01), marital status and age (p<0.05),
German language version	1021/1250 (81.7%)	a.2.79/2.67					
PACIC 5As	Patients with OA in primary care practices	b.3.51/3.39					
		c.3.34/3.33					
		d.2.41/2.31					
		e.2.39/2.29					
		f.2.94/2.62					
Rosemann [20]	Germany	a.2.44 (0.90)		Adequate variability in the overall scale & all subscales		PACIC and GS/T and FU/C scores significantly higher for patients with co-morbid diabetes, but no significant associations with other co-morbidities (hypertension, depression, CHD, COPD)	Age and gender showed weak correlations with overall PACIC and majority of subscales; no significant relationship with educational level.
German language version	Sample 1: 236/300 – 78.6%.	b.2.79 (0.85)		Floor effects in 3 subscales (F/C - 4.6%; PA - 3.8%; and GS/T - 3.4%).		Strong correlations found between PACIC sub scales and EUROPEP as expected	
PACIC 5As	Sample 2: 71 of subset of 75 sent follow up questionnaire after 2 weeks	c.2.56 (0.78)		Ceiling effects below 1.3%			
	OA patients from 75 primary care practices	d.2.31 (0.81)					
		e.2.48 (0.86)					
		f.2.01 (0.81)					
		(Test-retest - overall 0.81; PA 0.77; DSD/DS 0.78; GS/T 0.82; PS/C 0.79; FU/C 0.85.					
Schmittdiel [10]	USA	Mean 2.7		71% completed all items, 90% completed 17+		Relationships similar for subgroup by disease	Significant relationship with higher quality of life (OR 1.2); no relationship with adherence to medications (OR 1.06)
	4108/6673 (61%)					Higher ratings of health care (OR 2.36),	Significantly associated with greater engagement in self management behaviours (OR 1.21 to 1.41); use of self management services (OR 1.4)
	Private health care members on one of six chronic disease registers						
Szecenyi [21]	Germany	DMP/Non-DMP					Mean 3.2 DMP versus 2.68 non-DMP (significant p=0.001) and across all subscales except patient activation (p=0.05), greatest mean difference in F/C, least in PA
German language version	1532/3546 (42.2%)	a.3.26/2.86					
	(1,399 valid responses = 39%)	b.3.26/3.09					
PACIC 5As	Patients with type 2 diabetes in primary care, in or outside disease management programmes (DMPs)	c.3.52/3.29					
		d.2.91/2.50					
		e.3.39/3.04					
		f.3.13/2.70					
Taggart [14]	Australia	S 1	S 2	Sample 1: 73% completed all 20 items; 95% completed at least 17 items.	EFA, both 2 factor solutions, 59% & 61% variance	Higher PACIC scores associated with higher patient self-rated health	Degree/diploma, retired, hypertension/IHD & greater duration of disease had negative associations with both factors and total PACIC scores; Employed and married/CH had negative associations with planned care factor and total PACIC score
	Sample 1: 2552/2642 (96%) (2642 of 3349 asked & consented to take part)	a. 3.01	a.3.07	Sample 2: 79% completed all 20 items; 95% completed at least 17 items.	F1 SDM and SM (12 items across four scale) (alpha 0.939 & 0.943)	SDM and AM positively associated with good health	
					F2 Planned care (8 items across 3 scales) (Alphas 0.883 and 0.878)		
	Sample 2: 963/1000 (96%) (1000 out of 4167 consented to take part)						
	Patients with CHD, hypertension and/or T2 diabetes in general practice						
Wensing [22]	Netherlands	a. 2.9 (0.93)		22-35% missing data. Items 15, 17 & 20 had >30% non response	PCA – five factors	Association between PACIC and EUROPEP aggregated scores all positive as expected.	Higher enablement in patients associated with lower PACIC scores – contrary to expectations
Dutch language version	165 (72%)	b. 3.2 (0.85)		Lowest response category used by >30% for 11 items. (7-76%)	(70% variance explained; KMO 0.844; Bartlett’s p=0.000)		
	Randomly sampled patients with diabetes or COPD from four general practices (involved in a programme to enhance structured diabetes care)	c. 3.5 (0.75)		Highest response category used by >30% for 6 items (10 – 54%)	Matched three pre- defined domains (but not delivery system/practice design nor follow up/co-ordination)		
		d. 2.5 (0.81)					
		e. 3.3 (0.87)					
						

^1^PA = Patient activation ; DS/PD = Delivery system design; GS/T = Goal setting; PS/C = Problem solving; F/C = Follow up and Co-ordination; PHQ9 = Patient health Questionnaire; EUROPEP = European patient evaluation of general practice care; CFA = confirmatory factor analysis; EFA = exploratory factor analysis.

Validating scales such as the PACIC is a complex process. Although convergent validity with related scales (such as other patient self-report measures of quality) is useful [7,8], construct validity is difficult because it is not clear exactly how factors such as age, sex, socioeconomic status and multimorbidity should relate to PACIC scores. Studies have demonstrated predicted relationships with measures of self management behaviour [10,12] and self rated health [11,14]. Studies relating PACIC to ‘harder’ outcomes such as clinical parameters have generally had less success [9,13]. There are few prospective studies of the ability of PACIC to predict outcomes over time.

The published literature on the PACIC includes studies from the US [7,9,10,12,13,15,18], Canada [23], Denmark [11], Germany [8,19,21], Holland [22], Australia [14] and New Zealand [16]. Many findings are consistent across health systems and populations. However, at the time of writing there is little evidence about the performance of PACIC in the United Kingdom (UK), despite the major initiatives (such as the Quality and Outcomes Framework) which have been implemented in this setting to improve care for long-term conditions.

We present preliminary data on the psychometric performance of the PACIC in a large sample of UK patients with long-term conditions, in terms of acceptability, reliability and validity. For acceptability, we explored rates of missing data and compared them with rates found in the international literature. In terms of reliability, we assessed internal consistency at the scale and subscale level. In terms of validity, we explored floor and ceiling effects, factor structure, and associations between the PACIC and other care quality outcomes measures to test predicted relationships.

## Methods

Data were collected as part of a wider longitudinal cohort designed to assess the impact of ‘care planning’ and written ‘care plans’ on patient outcomes [24]. We identified patients on clinical registers with long-term conditions in practices with high levels of ‘care plans’ as reported in the General Practice Patient Survey [25], and recruited comparable patients in similar practices reporting lower levels of written care plans. The study was not designed to provide population estimates, but to create patient groups differing in rates of ‘care plans’ but similar in all other characteristics. However, the sample should function for assessment of psychometric characteristics and associations between variables. The current analysis uses baseline data from the cohort. The following measures were used.

### PACIC

As noted previously, the original version of PACIC used in the study includes 20 items based around five subscales: *patient activation*; *delivery system design*; *goal setting*; *problem-solving and contextual counselling*; and *follow-up and co-ordination*. Each item is rated on a five point scale (from ‘almost never’ to ‘almost always’) and subscale and total scores are based on average scores across items [7], with higher scores indicating higher quality of care. Item content is shown in Table 2. The scale was used without any major adaptation for a UK population, although ‘chronic condition’ was changed to ‘long-term condition’ as this is the more usual term used in the UK.

**Table 2:** Descriptive data on items and scales

**Item**	**Missing (%)**	**Floor/ceiling (% valid)**
Asked about my ideas when we made a treatment plan	13.0	
Given choices about treatment to think about	11.2	
Asked to talk about any problems with my medicines or their effects	9.6	
Given a written list of things I should do to improve my health	10.8	
Satisfied that my care was well organised	10.8	
Shown how what I did to take care of myself influenced my condition	14.1	
Asked to talk about my goals in care for my condition(s)	13.9	
Helped to set specific goals to improve my eating or exercise	13.8	
Given a copy of my treatment plan	15.6	
Encouraged to go to a specific group or class to help me cope with my long-term condition(s)	14.8	
Asked questions, either directly or on a survey, about my health habits	13.5	
Sure that my doctor or nurse thought about my values, beliefs and traditions when they recommended treatments to me	14.4	
Helped to make a treatment plan that I could carry out in my daily life	15.9	
Helped to plan ahead so I could take care of my condition(s) even in hard times	15.7	
Asked how my long-term condition(s) affects my life	14.5	
Contacted after a visit to see how things were going	14.9	
Encouraged to attend programs in the community that could help me	15.5	
Referred to a dietician or nutritionist	14.8	
Told how my visits with other types of doctors, like an eye doctor or surgeon, helped my treatment	14.8	
Asked how my visits with other doctors were going	14.1	
Patient activation scale	11.2	20.9%, 5.0%
Delivery system design subscale	12.4	3.7%, 5.0%
Goal setting subscale	14.0	14.2%, 1.3%
Problem solving subscale	15.7	14.7%, 5.1%
Follow up subscale	14.7	30.4%, 1.0%
PACIC total score	14.6	2.1%, 0.3%

#### Demographic and clinical characteristics

We measured socio-demographic variables (age, gender, work, and education). We asked patients to self report long-term conditions from a list (including high blood pressure, chest complaints, diabetes, heart problems, chronic kidney disease, stroke, cancer, anxiety and depression, arthritis, stomach or bowel problems, skin conditions, vision or hearing problems, neurological problems, chronic fatigue, thyroid or other problems). Patients also reported the professional they consulted with most frequently for their long-term conditions (GP, practice nurse or other, including community nurse, hospital doctor or hospital nurse) and the number of primary care consultations in the last six months.

### Measures of quality of care

#### (a) Shared decision making

We measured shared decision making using the Health Care Climate Questionnaire (HCCQ) measure [26,27]. The scale assesses patients’ perceptions of the degree to which their health professional is ‘autonomy supportive’ as opposed to ‘controlling’ when providing health care. Each item is scored on a 7-point scale ranging from ‘strongly disagree’ to ‘strongly agree’. We used the short form with 6 items, with an alpha of 0.8. Scale scores were recoded 0–100 for descriptive analysis, although there was evidence of significant skew.

#### (b) Quality of care for long-term conditions

We used a six item scale used in quality improvement activities in the UK which assess quality of care for long-term conditions with items relating to communication, patient involvement, information, support, co-ordination of care, and self-efficacy (*QIPP scale*). Each item is scored on a 4 point scale, with a range of scale anchors, and the item scores are averaged to create an overall score.

#### (c) Satisfaction with primary care

We assessed satisfaction with primary care with a single item 5 point scale (rated from ‘very dissatisfied’ to ’very satisfied’) [25]. Satisfaction data were very highly skewed.

### Analysis

#### (a) Acceptability

The PACIC scale did not go through any form of translation from US to UK populations. One indicator of the acceptability of a measure is to look at how well the items are completed. We assessed acceptability for the UK population by looking at completion rates and the extent of missing data. We computed missing data rates for items, sub-scales and overall score. There are no published guidelines for dealing with missing values on PACIC, so we adopted the arbitrary criterion that respondents must have completed at least 60% of the items on a subscale or the total scale to be included in analyses.

#### (b) Reliability

As with many scales, multiple items are used to measure PACIC subscales, on the basis that several observations will lead to a more reliable measure. This is based on the assumption that items within a subscale are homogenous. We assessed the internal consistency of the PACIC by calculating Cronbach’s alpha for the full PACIC scale and for each subscale.

#### (c) Validity

We calculated the proportions of patients scoring at floor and ceiling for subscales and the overall scale, and explored the distribution of subscale and overall scores.

Confirmatory factor analysis was used to test the hypothesised factor structure of the PACIC: a five latent-factor model of quality of care in which all latent factors were allowed to covary with one another [9]. Structural equation modelling, using AMOS (version 16.0), was used to fit and test the factor structure. We conducted two analyses. The first was a ‘complete cases’ analysis using only those respondents with full data on all 20 items; the second adopted a less restrictive criterion and included patients with missing data on three or fewer of the 20 items and no more than 50% of items missing on any of the five scales. STATA’s method of multivariate normal regression was used to impute ratings for these cases. As the imputed data was non-integer and, in a few cases, outside the item scoring range, it was first rounded to the nearest integer and then recoded, where necessary, to the appropriate ‘anchor’ point. The method of maximum likelihood was adopted for parameter estimation: asymptotically distribution-free estimation was employed as a sensitivity analysis.

The published evidence was mixed with respect to likely associations with demographic characteristics (see Table 1). We made no specific hypotheses, but report differences in the scores of different groups using linear regression (in Stata version 11.0), taking account of the within-practice clustering. Due to skewness in the distribution of the overall PACIC scores, standard errors were calculated using a bootstrap method, free from parametric assumptions, using 10,000 bootstrap samples.

To assess construct validity, we hypothesised significant associations with measures of shared decision-making, quality of care and satisfaction with primary care services (Table 1). We assessed these relationships using Spearman non-parametric correlations, in view of the skewed distributions in the measures.

## Results

Responses were received from 2551 respondents (41%), although a small number with missing age and sex were removed for analysis, as were those who self reported no long-term conditions (despite being on a clinical register), leaving 2439 potential respondents for analysis (40%). Demographic and clinical data on respondents are provided in Table 3.

**Table 3:** Descriptive data on the study sample

**Characteristic**		**Values are % or mean (SD)**
Gender	Male	51.3
Age	18 to 49	17.5
	50 to 64	30.8
	65 to 74	28.4
	75+	23.3
Self reported long-term conditions		
	One	20.5
	Two	28.3
	Three	20.5
	Four or more	30.6
Main professional responsible for care of long-term conditions*		
	Primary care (GP or nurse)	86.2
	Other	13.8
Highly satisfied with primary care		56.2
Shared decision making (HCCQ)		71.8 (26.2)
High rating of shared decision making (HCCQ)		48.1
Quality of care for long-term conditions (QIPP)		3.3 (0.67)

*9.4% of patients did not identify a ‘main professional.

### Acceptability

Missing data rates for the PACIC items were high (Table 2), ranging from 9.6% to 15.9% at an item level. Between 11.2% and 15.7% of subscales could not be calculated because less than 60% of items were completed and 14.6% of patients were missing a total score. Ceiling effects were generally under 5%, although significant proportions of patients scored at the floor for *patient activation* (20.9%), *goal setting* (14.2%), *problem solving* (14.7%) and *follow-up and co-ordination* (30.4%).

### Descriptives

The total PACIC score showed a reasonable distribution of scores, with some positive skew. Most of the subscales were also positively skewed, most notably the goal setting and follow up subscales. The mean overall PACIC score was 2.4 (SD 0.87) with subscale means of *patient activation* (2.5), *delivery system design* (3.1); *goal setting* (2.2); *problem solving* (2.5); and *follow-up and co-ordination* (1.9). The distribution of PACIC scores demonstrated more symmetry and less ceiling effects than the QIPP, HCCQ and satisfaction scores. Importantly, the distribution in the PACIC scores means the scale has much higher capacity to reflect positive changes in individual scores than the latter scales (see Additional files 1, 2, 3, 4, 5, 6, 7, 8 and 9).

The intracluster correlation coefficient (ICC) for the total PACIC score was 0.040 (i.e. only 4% of the total variation in PACIC scores was due to differences in practice means, with the remaining 96% resulting from differences between patients) with subscale ICCs ranging from 0.029 to −0.042.

### Reliability

Alpha reliabilities were as follows: *patient activation* (0.86, 3 items); *delivery system design* (0.68, 3 items); *goal setting* (0.82, 5 items); *problem solving* (0.86, 4 items); *follow-up and co-ordination* (0.82, 5 items); PACIC total (0.94, 20 items).

### Validity (structure)

The complete case analysis of the hypothesised PACIC factor structure utilised 75.7% of the sample (n = 1846). The model did not fit the data well according to most indices of fit (actual indices and conventional levels of ‘good’ fit are presented in Table 4). Although the Standardised Root Mean-Squared Residual indicated that on average, observed and predicted item variances and covariances were not too dissimilar, this masks a number of large differences on specific covariance terms. Inter-factor correlations were also generally high, ranging from 0.60 to 0.97 (between *delivery system design* and *goal setting*).

**Table 4:** Fit indices for the confirmatory factor analysis

		**Complete case data**		**Complete case plus imputed data**
	**Criterion for ‘Good’ Fit**	**Maximum likelihood**	**Asymptotically distribution-free**	**Maximum likelihood**
**N**		1,846	1,846	2,040
**χ**^**2**^**(d.f.)**	non-significant	3,535.3 (160)*	1,576.2 (160)*	3,895.3 (160)*
**Inter-factor correlation**		0.604 to 0.972	0.684 to 0.919	0.596 to 0.967
**NFI**	≥ .90	0.840	0.572	0.840
**CFI**	≥ .95	0.846	0.595	0.846
**RMSEA**	< .08	0.107	0.069	0.107
**SRMR**	< .08	0.068	0.092	0.068

*All three chi-squared statistics indicate a lack of fit of their respective model; however, in large samples, even trivial differences in model fit often cause chi-squared to be significant.

NFI. Normed Fit Index.

CFI.Comparative Fit Index.

RMSEA.Root Mean-Squared Error of Approximation.

SRMR.Standardised Root Mean-Squared Residual.

Using the less restrictive criteria a further subset of 194 patients (8%) with some missing data were added into the analysis, but the overall results in terms of indices of fit were similar (Table 4).

### Validity (construct)

The high inter-correlations between PACIC subscales and the failure to confirm a five factor structure meant that analyses of construct validity focused on PACIC total scores only. Initial analysis explored associations with demographic characteristics. Females and patients aged 75 or more scored significantly lower in total scores (regression co-efficient 0.18 and −0.20 respectively). The impact of increasing numbers of conditions and greater contact with a general practitioner was inconsistent. There was no association between scores and the professional most responsible for care of the long-term condition. All these relationships accounted for around 1% of the variance in PACIC scores (Table 5).

**Table 5:** Associations between PACIC scores and demographic variables

**External variable**	**N**	**Co-efficient**	**95% CI**	**% variance**
Female gender	1787	−0.18	−0.25 to −0.12	0.01
Age	1787			
up to 49		Reference	−0.19 to 0.08	0.01
50–64		−0.05	−0.19 to 0.12	
65–74		−0.03	−0.36 to −0.04	
75+		−0.20		
Conditions	1787			
1		Reference		0.01
2		−0.04	−0.18 to 0.11	
3		−0.16	−0.32 to −0.01	
		−0.12	−0.27 to 0.04	
4+				
Main professional responsible for long-term condition not primary care	1652	−0.01	−0.11 to 0.10	0.00
GP visits in 6 months	1787			0.00
0		Reference		
1		0.03	−0.09 to 0.15	
2		0.12	−0.01 to 0.25	
3		0.14	0.02 to 0.28	
4+		0.11	−0.03 to 0.25	

In terms of construct validity (Table 6), PACIC total scores were significantly associated with the single item measure of patient satisfaction with primary care (Spearman’s correlation 0.24) and demonstrated higher correlations with shared decision-making (Spearman’s correlation 0.47) and quality of care (Spearman’s correlation 0.54).

**Table 6:** Associations with other self-reported measures of care

**Variable**	**N**	**Spearman rank correlation**	**p**
Satisfaction with primary care	1827	0.24	<0.001
Shared decision making (HCCQ)	1780	0.47	<0.001
Quality of care for long-term conditions (QIPP)	1817	0.54	<0.001

The results were not markedly different when the analyses were rerun on the imputed data set (N = 1973).

## Discussion

As health policy makers focus on the challenges of care for long-term conditions, significant funding is being channelled towards quality improvement in care delivery, through changes to skill mix, staff training, new technologies, and financial incentives. The success of these quality improvement efforts are in part dependent on effective measures to track current standards and assess the effectiveness of interventions. Such measures can also ensure that policy and clinical interventions are perceived by patients to be making improvements to care. This study represented a preliminary test of the utility of the PACIC for this purpose in the UK.

### Summary of the results

Scores on the PACIC showed some skew, but were generally reasonably well distributed, with few scales showing the high levels of skew that that are sometimes evident on other patient-reported measures of primary care. However, the amount of missing data at the item, subscale and overall levels was relatively high. This was higher than comparable PACIC studies in the literature, where rates (when reported) were around 3–5% [7-9,11]. It was also higher than rates found in scales in the same survey – for example, the shorter QIPP had missing data rates of 3–5% at an item level.

### Limitations of the study

As noted earlier, the study was designed as a longitudinal study to assess the potential benefits of care plans. The sample was not designed as a random sample of patients with long-term conditions, and the response rate, while in line with other published studies [28], does mean that the sample cannot be considered representative. It should function as a sample for preliminary assessment of the performance of the scale, although selective non-response (i.e. among more severely ill patients) may restrict range on some variables, which could in turn impact on estimated associations. Furthermore, care must be taken in interpreting descriptive data such as mean scores.

We did not have data to estimate some important aspects of reliability and validity, including test-retest reliability, criterion validity (as there is no accepted ‘gold standard’) or responsiveness to change. Our assessment of acceptability was limited to missing item rates, and did not explore other aspects, such as patient views of the scale, time to complete the scale, or cultural acceptability [29].

### Interpretation of the results

It is not clear why non-completion rates were so much higher than comparable studies, and there are a lack of data to provide comparisons of patient characteristics (such as education and health literacy) in the current sample which might explain these high rates. Examination of the item content of the PACIC might suggest that some phrases (such as ‘nutritionist’) may be unfamiliar to some patients, and others (such as ‘hard times’) may be interpreted differently between UK and other populations. Informal discussions took place with some patients during administration of the survey, and those discussions suggested that some items on PACIC may make assumptions about existing care in the UK which may be inappropriate. For example, question 1 is ‘Asked about my ideas when we made a treatment plan’, and question 8 asks patients if they were ‘given a copy of my treatment plan’. The items represent *reports* of care, but the response options do not offer a ‘not relevant’ option, hence it is possible that some ‘missing’ responses reflect patients who felt that the question was *irrelevant* to their current care, rather than just representing activities that were infrequent, as evidence suggests that written treatment plans are not a consistent part of care for long-term conditions in the UK [24]. The current response format may be causing missing data which in fact reflects meaningful responses. It has been suggested that response scales may reasonably be modified to suit local context and this might improve performance of the scale in the UK, although this will be at some potential cost in comparability across studies [30]. This issue requires further investigation and possibly cognitive testing and other qualitative methods to make the scale more suitable for the UK population.

If the scores of respondents can be considered meaningful, it is interesting that the scores on PACIC in the UK are relatively low. Some scales did show a high prevalence of scores at the floor, and the mean scores were generally lower than those reported in the wider literature. For example, mean PACIC total score was 2.4, compared to 2.6 in patients in US primary care [7], 3.3 in depressed primary care patients in Germany [8], 2.7 in patients with osteoarthritis in German primary care [19], 3.0 in patients with CHD, hypertension or diabetes in Australian general practice [14] and 3.2 in Hispanics with diabetes in hospital ambulatory settings in the US [15]. *Patient activation*, *follow-up and co-ordination* and *problem solving* were particularly low in the current sample. Of course, there are a lack of data on calibration of PACIC against other measures which would allow judgements of the clinical or policy significance of such differences, even if they were statistically significant. However, the low scores may seem surprising given the importance placed on structured delivery of care for long-term conditions through the Quality and Outcomes Framework, which has seen changes to skill mix, and increased use of information technology and protocols for monitoring patients and delivering standardised care in line with the Chronic Care Model [31]. Recent evidence suggests that patients with complex care needs in the UK rate their experience of a ‘patient-centered medical home’ (characterised by high access, professionals who know their medical history, and care coordination) higher than those in other countries [32]. However, there is evidence that the content of care has changed, with an increased focus on biomedicine and less on self management and psychosocial issues [33-35], and it is possible that the scores reflect that.

Generally, the PACIC subscales showed appropriate levels of internal reliability. We did not set an a priori criterion for reliability prior to the analysis, although our implicit assumption was that they should be between 0.7 and 0.9, in line with published convention [29]. Cronbach’s alpha for *Delivery system design* was lower than for the other subscales (0.68 vs. >0.80). It should be noted that this pattern is consistent with data from other studies where reported [7,8,22] and as these other studies are from the USA, Germany and Holland, the lower reliability seems unlikely to reflect the UK health service.

As indicated in Table 1, studies have reported variable relationships with demographic and clinical variables. We found lower PACIC scores in females, while the bulk of studies report non-significant relationships [8,11,12,15], although that may reflect the higher level of power in the current analysis as the proportion of variance accounted for by gender was trivial. The same patterns were in evidence for relationships with age [8,11,12,15,20] and number of conditions [7,8,11,12,15].

In terms of validity, the PACIC showed the hypothesised associations with shared decision-making and assessments of quality of care and patient satisfaction. Global measures of satisfaction generally reflect patient assessments of interpersonal care, and it appears that PACIC is not simply reflecting the quality of the doctor-patient relationship or patients’ liking for their doctor, as the associations are relatively low. The different distributions of scores indicate that PACIC has the potential to add value to the assessment of practice and professional performance.

The factor analysis suggested that the five factor structure was not supported by the data. Although further analysis might formally test alternative models of the relationships between items, calculating total PACIC scores based on all 20 items might be the most appropriate scoring method. It should be noted that maximum likelihood estimation is not considered to be the best method for use with ordinal data [36], as it was developed for continuous variables with a joint multivariate normal distribution. However, the large sample size, coupled with the knowledge that we are following applied measurement practice for this instrument (i.e. item scores are simply summed to form subscale and overall scores) justify its use here.

Some previous analyses have supported the five factor structure [7,20,22], although technical aspects of these analyses have been criticised [30]. Of course, as this is the first published assessment of the PACIC scale in the UK, the failure to confirm the factor structure may reflect characteristics of the service context and the patient population, such as the gap between the assumptions inherent in PACIC items and the experience of patients that was raised in the discussion of missing data above. If patient experience of care for long-term conditions is not effectively reflected in the PACIC items, a clear factor structure may be less likely to emerge.

More fundamentally, the appropriateness of factor analysis (and internal reliability estimates) has been questioned. Underlying these techniques is the assumption that responses to individual items are caused by an underlying construct [37]. Patients reporting inconsistent patterns across related items may not reflect instrument problems, but inconsistency in their experience of separate and distinct aspects of care. If this is the case, conventional assessments of factor structure and internal reliability may be less useful [30].

Although data are available in the baseline cohort, we have not reported associations between PACIC and patient health behaviour and health outcomes. We do not feel that these are correctly conceptualised as measures of validity for a single scale: rather, the association between quality of care and patient outcomes (and the importance of care quality compared to other drivers such as demography, socio-economic status and self-management behaviour) is a core empirical question for health services research and delivery [38]. The priority is to explore whether quality of care predicts outcomes over time, where evidence is far more limited. Our longitudinal survey is designed to allow this to be estimated prospectively and we will publish data in due course.

## Conclusions

In summary, the study suggests that the use of PACIC may lead to relatively high levels of missing data among UK patients, although the reasons for that would benefit from further research. However, our analyses suggest reasonable levels of reliability and validity. The instrument also demonstrates a more symmetrical distribution than most patient-reported measures and a higher capacity to capture positive change, giving the scale (and the modified version currently proposed) considerable potential as a measure of the delivery of core components of care for long-term conditions in the UK.

## Competing interests

The authors declare that they have no competing interests.

## Authors’ contributions

PB, MR, BS and DR are applicants on the grant. JR and KR conducted the survey, with PB. MH conducted the analyses. PB and JR wrote the paper and all other authors commented on earlier drafts. All authors read and approved the final manuscript.

## Pre-publication history

The pre-publication history for this paper can be accessed here:

http://www.biomedcentral.com/1472-6963/12/293/prepub

## Supplementary Material

Additional file 1 **Figure S1. **Distribution of PACIC total scores. Click here for file

Additional file 2 **Figure S2. **Distribution of PACIC patient activation scores. Click here for file

Additional file 3 **Figure S3. **Distribution of PACIC Delivery system design scores. Click here for file

Additional file 4 **Figure S4. **Distribution of PACIC Goal setting scores. Click here for file

Additional file 5 **Figure S5. **Distribution of PACIC Problem solving scores. Click here for file

Additional file 6 **Figure S6. **Distribution of PACIC follow up scores. Click here for file

Additional file 7 **Figure S7. **Distribution of HCCQ scores scores. Click here for file

Additional file 8 **Figure S8. **Distribution of QIPP scores. Click here for file

Additional file 9 **Figure S9. **Distribution of single item patient satisfaction scores. Click here for file

## References

[B1] Wagner E Chronic disease management: What will it take to improve care for chronic illness? Effective Clinical Practice 1998 1 2 4 10345255

[B2] Department of Health Supporting people with long term conditions: An NHS and social care model to support local innovation and integration 2005 London

[B3] Kennedy A Rogers A Bower P Support for self care for patients with chronic disease BMJ 2007 335 968 970 10.1136/bmj.39372.540903.94 17991978 PMC2071971

[B4] Murray E Burns J See Tai S Lai R Nazareth I Interactive Health Communication Applications for people with chronic disease Cochrane Database of Systematic Reviews 2005 Issue 4. Art. No. CD004274 10.1002/14651858 PMC13184810 16235356

[B5] Campbell S Reeves D Kontopantelis E Sibbald B Roland M Effects of pay for performance on the quality of primary care in England N Engl J Med 2009 361 368 378 10.1056/NEJMsa0807651 19625717

[B6] Campbell S Roland M Buetow S Defining quality of care Soc Sci Med 2000 51 1611 1625 10.1016/S0277-9536(00)00057-5 11072882

[B7] Glasgow R Wagner E Schaefer J Mahoney L Reid R Greene S Development and validation of the Patient Assessment of Chronic Illness Care Med Care 2005 43 436 444 10.1097/01.mlr.0000160375.47920.8c 15838407

[B8] Gensichen J Serras A Paulitsch M Rosemann T König J Gerlach F Petersen J The Patient Assessment of Chronic Illness Care questionnaire: evaluation in patients with mental disorders in primary care Community Ment Health J 2011 47 453 10.1007/s10597-010-9340-2 20734231

[B9] Gugiu C Coryn C Applegate B Structure and measurement properties of the Patient Assessment of Chronic Illness Care instrument J Eval Clin Pract 2010 16 509 516 20210824 10.1111/j.1365-2753.2009.01151.x

[B10] Schmittdiel J Mosen D Glasgow R Hibbard J Remmers C Bellows J Patient assessment of chronic illness care (PACIC) and improved patient-centered outcomes for chronic conditions J Gen Intern Med 2011 23 77 80 18030539 10.1007/s11606-007-0452-5 PMC2173922

[B11] Maindal H Sokolowski I Vedsted P Adaptation, data quality and confirmatory factor analysis of the Danish version of the PACIC questionnaire Eur J Public Health 2010 10.1093/eurpub/ckq188 21134901

[B12] Glasgow R Whitesides H Nelson C King D Use of the Patient Assessment of Chronic Illness Care (PACIC) with diabetic patients Diabet Care 2005 28 2655 2661 10.2337/diacare.28.11.2655 16249535

[B13] Gugiu P Coryn C Clark R Kuehn A Development and evaluation of the short version of the Patient Assessment of Chronic Illness Care instrument Chronic Illness 2009 5 268 276 10.1177/1742395309348072 19933249

[B14] Taggart J Chan B Jayasinghe U Christl B Proudfoot J Crookes P Beilby J Black D Harris M Patients Assessment of Chronic Illness Care (PACIC) in two Australian studies: structure and utility J Eval Clin Pract 2011 17 215 221 10.1111/j.1365-2753.2010.01423.x 20846281

[B15] Aragones A Schaefer E Stevens D Gourevitch M Glasgow R Shah N Validation of the Spanish translation of the Patient Assessment of Chronic Illness Care (PACIC) survey Preventing Chronic Disease 2008 5 1 10 PMC2578783 18793501

[B16] Carryer J Budge C Hansen C Gibbs K Providing and receiving self-management support for chronic illness: Patients' and health practitioners' assessments Journal of Primary Health Care 2010 2 124 129 20690302

[B17] Goetz K Freund T Gensichen J Miksch A Szecsenyi J Steinhauser J Adaptation and psychometric properties of the PACIC Short Form Am J Manag Care 2012 18 e55 e60 22435885

[B18] Jackson G Weinberger M Hamilton N Edelman D Racial/ethnic and educational-level differences in diabetes care experiences in primary care Primary Care Diabetes 2008 2 39 44 10.1016/j.pcd.2007.11.002 18684419

[B19] Rosemann T Laux G Szecsenyi J Grol R The Chronic Care Model: congruency and predictors among primary care patients with osteoarthritis Qual Saf Health Care 2008 17 442 446 10.1136/qshc.2007.022822 19064660

[B20] Rosemann T Laux G Droesemeyer S Gensichen J Szecsenyi J Evaluation of a culturally adapted German version of the Patient Assessment of Chronic Illness Care (PACIC 5A) questionnaire in a sample of osteoarthritis patients J Eval Clin Pract 2007 13 806 813 10.1111/j.1365-2753.2007.00786.x 17824876

[B21] Szecsenyi J Rosemann T Joos S Peters-Klimm F Miksch A German diabetes disease management programs are appropriate for restructuring care according to the chronic care model Diabet Care 2011 31 1150 1154 10.2337/dc07-2104 18299443

[B22] Wensing M van Lieshout J Jung H Hermsen J Rosemann T The Patient Assessment Chronic Illness Care (PACIC) questionnaire in The Netherlands: a validation study in rural general practice BMC Health Services Research 2008 8 182 10.1186/1472-6963-8-182 18761749 PMC2538520

[B23] McIntosh C *Examining the factorial validity of selected modules from the Canadian Survey of Experiences with Primary Health Care (http://www.statcan.ca).* Ottowa; 2011

[B24] Burt J Roland M Paddison C Reeves D Campbell J Abel G Bower P Use and benefits of care plans and care planning for people with long-term conditions in England J Health Serv Res Policy 2012 17 64 71 10.1258/jhsrp.2011.010172 22315479

[B25] Campbell J Smith P Nissen S Bower P Elliott M Roland M The GP Patient Survey for use in primary care in the National Health Service in the UK - development and psychometric characteristics BMC Family Practice 2009 10 10.1186/1471-2296-10-57 PMC2736918 19698140

[B26] Williams G McGregor H Zeldman A Freedman Z Deci E Testing a self-determination theory process model for promoting glycemic control through diabetes self-management Health Psychol 2004 23 58 66 14756604 10.1037/0278-6133.23.1.58

[B27] Williams G McGregor H Kind D Nelson C Glasgow R Variation in perceived competence, glycemic control, and patient satisfaction: relationship to autonomy support from physicians Pat Educ Couns 2005 57 39 45 10.1016/j.pec.2004.04.001 15797151

[B28] Roland M Elliott M Lyratzopoulos G Barbiere J Parker R Smith P Bower P Campbell J Reliability of patient responses in pay for performance schemes: analysis of national General Practitioner Patient Survey data in England BMJ 2009 339 b3851 10.1136/bmj.b3851 19808811 PMC2754504

[B29] Fitzpatrick R Davey C Buxton M Jones D Evaluating patient-based outcome measures for use in clinical trials Health Technol Assess 1998 2 14 9812244

[B30] Spicer J Budge C Carryer J Taking the PACIC back to basics: the structure of the Patient Assessment of Chronic Illness Care J Eval Clin Pract 2010 6 10.1111/j.1365-2753.2010.01568.x 20973876

[B31] Maisey S Steel N Marsh R Gillam S Fleetcroft R Howe A Effects of payment for performance in primary care: qualitative interview study J Health Serv Res Policy 2008 13 133 139 10.1258/jhsrp.2008.007118 18573761

[B32] Schoen C Osborn R Squires D Doty M Pierson R Applebaum S New 2011 survey of patients with complex care needs in eleven countries finds that care is often poorly coordinated Health Aff 2012 30 2437 2448 10.1377/hlthaff.2011.0923 22072063

[B33] Checkland K Harrison S McDonald R Grant S Campbell S Guthrie B Biomedicine, holism and general medical practice: responses to the 2004 General Practitioner contract Sociology of Health and Illness 2008 30 788 803 10.1111/j.1467-9566.2008.01081.x 18444956

[B34] Blakeman T MacDonald W Bower P Gately C Chew-Graham C A qualitative study of GPs' attitudes to self-management of chronic disease Br J Gen Pract 2006 56 407 414 16762121 PMC1839014

[B35] MacDonald W Rogers A Blakeman T Bower P Practice nurses and the facilitation of self-management in primary care J Adv Nurs 2008 62 191 199 10.1111/j.1365-2648.2007.04585.x 18394031

[B36] Flora D Curran P An empirical evaluation of alternative methods of estimation for confirmatory factor analysis with ordinal data Psychological Methods 2004 9 466 491 15598100 10.1037/1082-989X.9.4.466 PMC3153362

[B37] Bollen K Lennox R Conventional wisdom on measurement: a structural equation perspective Psychol Bull 1991 110 305 314

[B38] Kahn K Tisnado D Adams J Liu H Chen W Hu F Mangione C Hays R Danberg C Does ambulatory process of care predict health-related quality of life outcomes for patients with chronic disease? Health Serv Res 2007 42 63 83 10.1111/j.1475-6773.2006.00604.x 17355582 PMC1955244

