# Supplementary figures and images for: Psychometric properties of the patient assessment of chronic illness care measure: acceptability, reliability and validity in United Kingdom patients with long-term conditions
Source: BMC Health Serv Res. 2012 Aug 31;12:293. doi: 10.1186/1472-6963-12-293 (PMC3526462; doi:10.1186/1472-6963-12-293)

**Figure S1 Distribution of PACIC total scores**


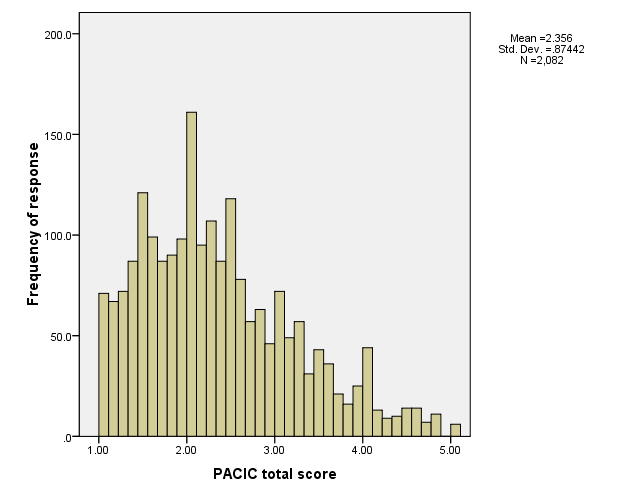

Supplement: Additional file 1 — Figure S1. Distribution of PACIC total scores. [file 1472-6963-12-293-S1.doc]

**Figure S2 Distribution of PACIC patient activation scores**


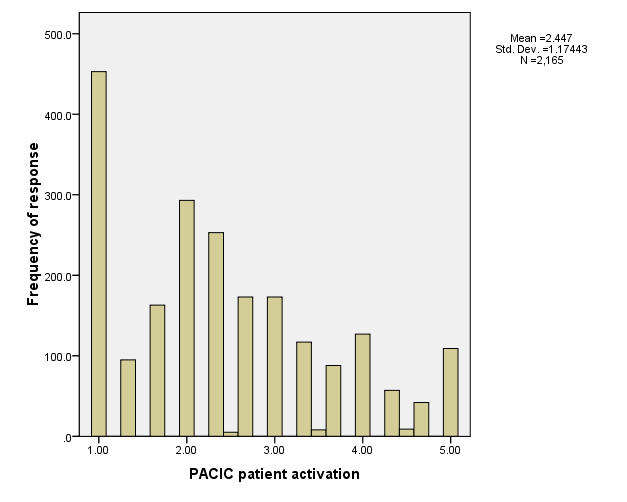

Supplement: Additional file 2 — Figure S2. Distribution of PACIC patient activation scores. [file 1472-6963-12-293-S2.doc]

**Figure S3 Distribution of PACIC Delivery system design scores**


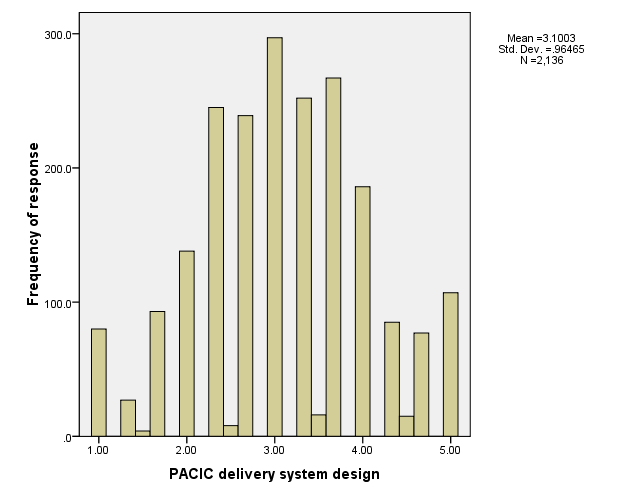

Supplement: Additional file 3 — Figure S3. Distribution of PACIC Delivery system design scores. [file 1472-6963-12-293-S3.doc]

**Figure S4 Distribution of PACIC Goal setting scores**


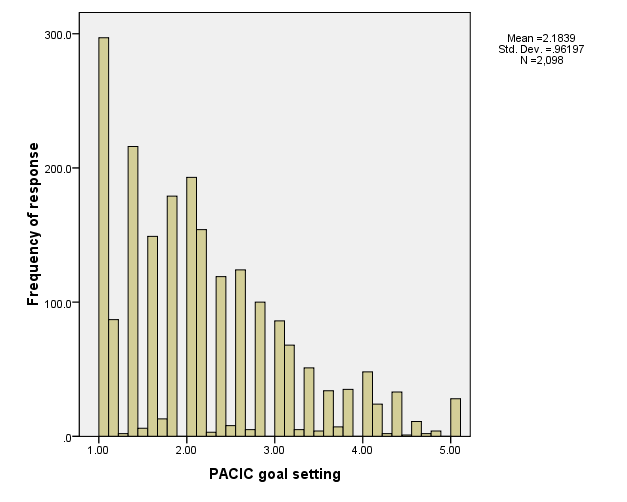

Supplement: Additional file 4 — Figure S4. Distribution of PACIC Goal setting scores. [file 1472-6963-12-293-S4.doc]

**Figure S5 Distribution of PACIC Problem solving scores**


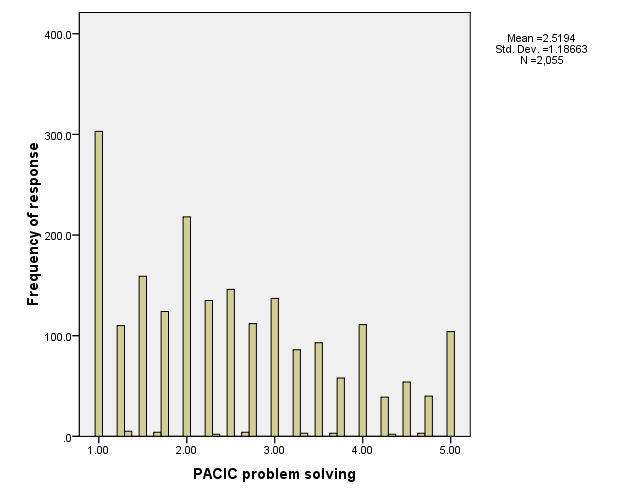

Supplement: Additional file 5 — Figure S5. Distribution of PACIC Problem solving scores. [file 1472-6963-12-293-S5.doc]

**Figure S6 Distribution of PACIC follow up scores**


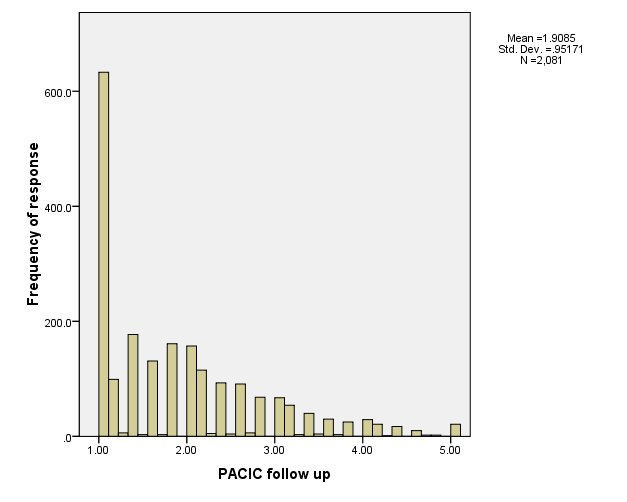

Supplement: Additional file 6 — Figure S6. Distribution of PACIC follow up scores. [file 1472-6963-12-293-S6.doc]

**Figure S7 Distribution of HCCQ scores scores**


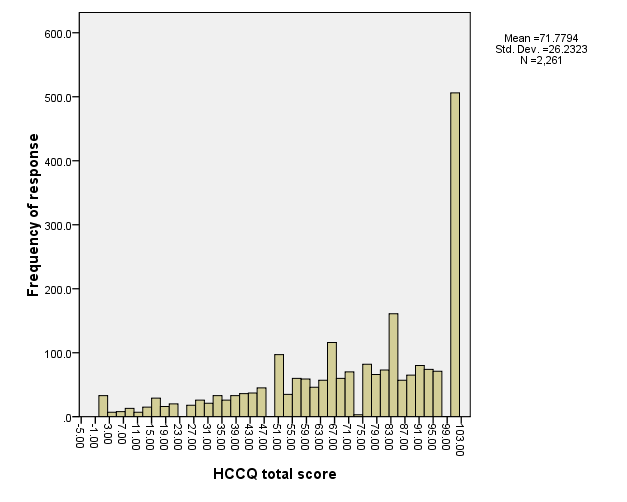

Supplement: Additional file 7 — Figure S7. Distribution of HCCQ scores scores. [file 1472-6963-12-293-S7.doc]

**Figure S8 Distribution of QIPP scores**


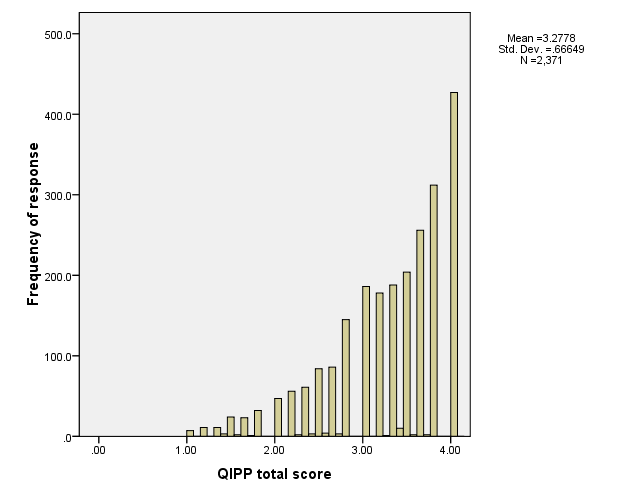

Supplement: Additional file 8 — Figure S8. Distribution of QIPP scores. [file 1472-6963-12-293-S8.doc]

**Figure S9 Distribution of single item patient satisfaction scores**


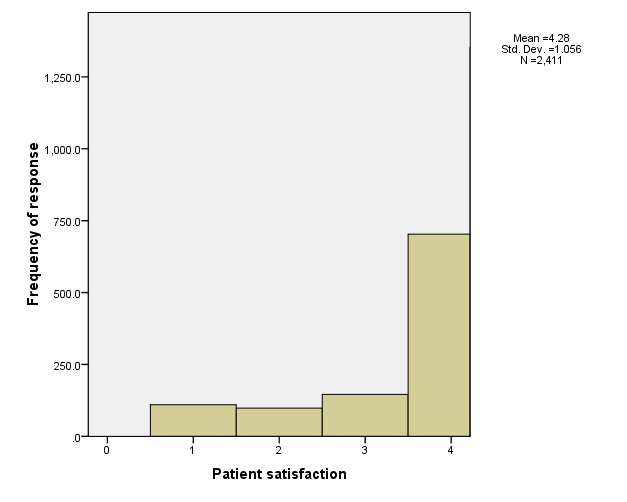

Supplement: Additional file 9 — Figure S9. Distribution of single item patient satisfaction scores. [file 1472-6963-12-293-S9.doc]
